# Effect of Aerosolization and Drying on the Viability of *Pseudomonas syringae* Cells

**DOI:** 10.3389/fmicb.2018.03086

**Published:** 2018-12-18

**Authors:** Malin Alsved, Stine Holm, Sigurd Christiansen, Mads Smidt, Bernadette Rosati, Meilee Ling, Thomas Boesen, Kai Finster, Merete Bilde, Jakob Löndahl, Tina Šantl-Temkiv

**Affiliations:** ^1^Ergonomics and Aerosol Technology, Department of Design Sciences, Lund University, Lund, Sweden; ^2^NanoLund, Lund University, Lund, Sweden; ^3^Department of Physics and Astronomy, Stellar Astrophysics Centre, Aarhus University, Aarhus, Denmark; ^4^Microbiology Section, Department of Bioscience, Aarhus University, Aarhus, Denmark; ^5^Atmospheric Physical Chemistry, Department of Chemistry, Aarhus University, Aarhus, Denmark; ^6^Department of Molecular Biology and Genetics, Aarhus University, Aarhus, Denmark

**Keywords:** bioaerosols, aerosolization, *Pseudomonas syringae*, drying, bubble bursting, ice nucleation activity

## Abstract

Airborne dispersal of microorganisms influences their biogeography, gene flow, atmospheric processes, human health and transmission of pathogens that affect humans, plants and animals. The extent of their impact depends essentially on cell-survival rates during the process of aerosolization. A central factor for cell-survival is water availability prior to and upon aerosolization. Also, the ability of cells to successfully cope with stress induced by drying determines their chances of survival. In this study, we used the ice-nucleation active, plant pathogenic *Pseudomonas syringae* strain R10.79 as a model organism to investigate the effect of drying on cell survival. Two forms of drying were simulated: drying of cells in small droplets aerosolized from a wet environment by bubble bursting and drying of cells in large droplets deposited on a surface. For drying of cells both in aerosol and surface droplets, the relative humidity (RH) was varied in the range between 10 and 90%. The fraction of surviving cells was determined by live/dead staining followed by flow cytometry. We also evaluated the effect of salt concentration in the water droplets on the survival of drying cells by varying the ionic strength between 0 and 700 mM using NaCl and sea salt. For both aerosol and surface drying, cell survival increased with decreasing RH (*p* < 0.01), and for surface drying, survival was correlated with increasing salt concentration (*p* < 0.001). Imaging cells with TEM showed shrunk cytoplasm and cell wall damage for a large fraction of aerosolized cells. Ultimately, we observed a 10-fold higher fraction of surviving cells when dried as aerosol compared to when dried on a surface. We conclude that the conditions, under which cells dry, significantly affect their survival and thus their success to spread through the atmosphere and colonize new environments as well as their ability to affect atmospheric processes.

## Introduction

Burrows et al. (2009) estimated that ∼10^24^ bacteria are emitted to the atmosphere each year on a global scale, and the quest to understand their contribution to microbial biogeography, gene flow, atmospheric processes, human health, transmission of diseases and agriculture has intensified during the past two decades (Polymenakou, 2012). Numerous studies report that the atmosphere serves as a major vector for the transfer of cells between bacterial communities (Griffin et al., 2003; Herbold et al., 2014; Šantl-Temkiv et al., 2018). Atmospherically dispersed bacteria may affect the assembly and genetic diversity of their new host communities through competition and horizontal gene transfer (Nemergut et al., 2013). In addition, airborne bacteria may also impact atmospheric processes. Due to their efficient ice nucleation activity, some bacterial species could for example impact the atmospheric water cycle by fostering ice formation in clouds, which is a major process involved in rain formation (Morris et al., 2008).

Studying microbial aerosolization is fundamental to the understanding of the airborne microbial dispersion between humans, animals, plants and different terrestrial and marine environments (Marthi et al., 1990; Walter et al., 1990; Heidelberg et al., 1997). Mechanical forces and wind-induced aerosolization from soil and plant surfaces are principal mechanisms generating atmospheric bioaerosols from dry terrestrial surfaces (Jones and Harrison, 2004). Wind-induced wave breaking followed by bubble bursting is the main aerosolization mechanism from marine and freshwater surfaces (Blanchard and Syzdek, 1970). Bubble bursting can also be important in a terrestrial context, as Joung et al. (2017) recently found that bubbles formed by raindrops splashing on soil surfaces can aerosolize microbial cells, which is a process that may contribute up to 25% of the global bacterial emissions. Finally, bacteria in urban and indoor environments are released from water surfaces by bubble bursting, e.g., from wastewater treatment plants, washrooms and sinks. Bubble bursting produces two kinds of droplets: film droplets released in high numbers (up to 1000 per bubble) from the bursting film, and jet droplets released vertically upwards in limited numbers (up to 10 per bubble). Both film drops, with typical diameters of 1–10 μm, and jet drops, which are bigger with diameters of 6–100 μm (Löndahl, 2014), are large enough to carry bacteria.

Virtually all airborne cells are subject to drying either already in the terrestrial microenvironment, from where they get aerosolized, or in the aerosolized picolitre-sized droplets that are produced by bubble bursting. Drying is the major stress for airborne bacterial cells and may strongly diminish their survival probability both during aerosolization and transport (Potts, 1994). Despite the potential influence of atmospheric bacteria on ecosystem structure and function, our knowledge on how drying during the process of aerosolization affects the survival, and thus, dispersal probability of bacterial cells is limited.

Studies addressing the airborne dispersal of bacteria are few and there is no consensus on how cell viability is affected by aerosolization and prolonged airborne state (Cox, 1971; Marthi et al., 1990; Walter et al., 1990; Reponen et al., 1997; Tang, 2009). This discordance is to a large extent due to the complexity of experiments and the varying methodologies. Among the few available studies are Marthi et al. (1990) and Walter et al. (1990), who demonstrated that the survival of *P. syringae* cells during aerosolization depends on environmental conditions such as temperature and relative humidity (RH), salt concentration and the aerosol droplet size. In addition, several studies have demonstrated increased death rates of airborne Gram negative bacteria at intermediate RH (50–70%) to high RH (70–90%) (Dunklin and Puck, 1948; Webb, 1959; Cox, 1966, 1971; Won and Ross, 1966). These studies all relied on cultivation as a measure of cell survival. However, cells that are in the viable-but-non-cultivable (VBNC) state are common among aerosolized bacteria (Heidelberg et al., 1997) and it is thus essential to use cultivation-independent techniques to distinguish between live and dead cells. Recently, rapid single-cell analysis techniques became available, which promote comprehensive analysis of cellular states. For example, flow cytometry is a technique that could substitute cultivation-based methods, as it allows for rapid and accurate *in situ* analysis of single cell status (Khan et al., 2010).

In this study, we simulated two types of drying associated with aerosolization: (i) drying of cells in airborne picolitre-sized droplets generated by bubble bursting and (ii) drying of cells in microlitre-sized droplets dried on a surface. These types of drying stresses are characteristic for two major types of bacterial aerosolization: emissions from liquid environments and emissions from dry solid surfaces. The aims were firstly, to investigate the quantitative effect of cell drying on bacterial survival, and secondly, to understand how bacterial response to drying depends on different RH in air and ionic strength of the solution.

## Materials and Methods

### Bacterial Strain and the Culture Conditions

We used the ice nucleation active *Pseudomonas syringae* strain R10.79, isolated from rain (Šantl-Temkiv et al., 2015), as a model organism for this study. *P. syringae* are found in a wide range of aquatic, plant-surface, soil, and atmospheric environments (Morris et al., 2008; Monteil et al., 2012; Hill et al., 2014; Šantl-Temkiv et al., 2015) with several strains being opportunistic human or plant pathogens. Thus, we have selected *Pseudomonas syringae* strain R10.79 as a model that would allow us to understand which factors affect the potential airborne transfer of Gram-negative bacterial cells in general, and *Pseudomonas* species in particular. Liquid cultures of *P. syringae* strain R10.79 were grown in R2A liquid medium until stationary phase was reached. The cultures were centrifuged at 13,000 × *g* for 10 min, and the pellets were suspended in solutions corresponding to the individual experiment (MilliQ water, 0.1 wt% NaCl or 0.9 wt% NaCl and 0.5, 1.0, 1.5, 2.0, 2.5, or 3.5 wt% sea salt). All experiments were run in triplicates using independently grown stationary phase bacterial cultures. Some experiments were repeated a second time, yielding six replicates.

### Surface Drying of Bacterial Suspensions

**FIGURE 1:**
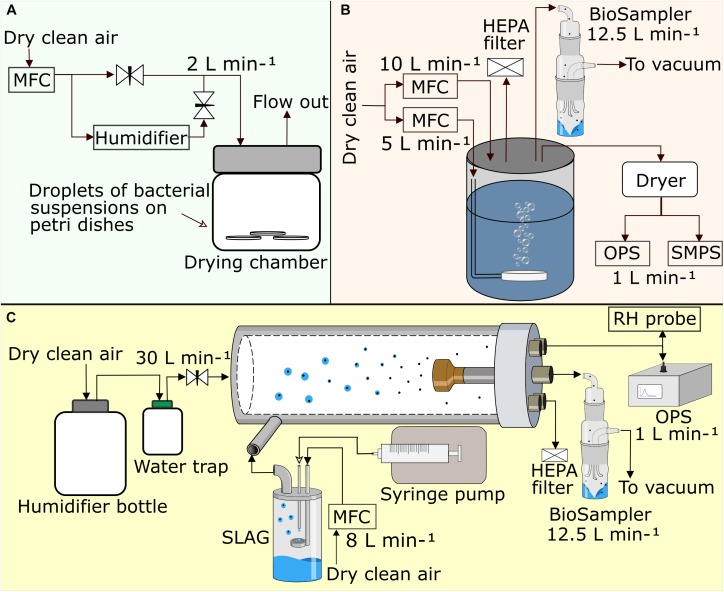
Schematic drawing of the three experimental setups for **(A)** surface drying in air with controlled RH, **(B)** aerosolization in bubble tank, and **(C)** aerosolization by SLAG into humidity conditioned flow tube. Black arrows show airflow directions. MFC, mass flow controller; OPS, optical particle sizer; SMPS, scanning mobility particle sizer; SLAG, sparging liquid aerosol generator; RH probe, relative humidity probe; HEPA filter, high efficiency particulate arresting filter.

To simulate drying of bacteria on terrestrial surfaces, which could be subsequently followed by wind-driven aerosolization, droplets of bacterial suspensions were dried on sterile Petri dishes (Sarstedt AG & Co.) in three experiments. In the first experiment, cells were suspended in solutions of different salinity and then dried in a sterile flow bench. In the second experiment, droplets containing bacterial cells were dried in air of different but constant RH (Figure 1A) by passing humidity-controlled air at a rate of 2 L min^-1^ (set by a thermal mass flow controller, MFC, Vögtlin Instruments, red-y smart series) through a drying chamber (volume 500 mL). The RH (15, 30, 60, or 80%, respectively) was continuously monitored in the outflowing air (Rotronic HC2-S, LOD: 1% RH, Accuracy ± 0.8%). In both experiments, six 25 μL droplets of a bacterial cell suspension, were placed on each Petri dish. The droplets contained ∼10^8^ cells mL^-1^. For droplet drying with different salt concentrations we used: 0.1 wt% and 0.9 wt% NaCl and 0.5, 1.0, 1.5, 2.0, 2.5, and 3.5 wt% sea salt (Sigma-Aldrich, S9883, 55% Cl, 31% Na, 8% SO_4_^2-^, 4% Mg, 1% K, 1% Ca, <1% other). In the third experiment, different droplet sizes (1, 5, 10, 25, and 75 μL) were dried in a sterile flow bench. For all droplet sizes, a total volume of 150 μL was dispatched into droplets. For example, 150 droplets of 1 μL and 15 droplets of 10 μL were prepared. In the second and the third experiment, cells were dried in 0.1 wt% NaCl solution.

Dried cells were washed off from the surface by repeatedly pipetting the same salt solution, which was initially used for drying, up and down until cells were suspended. Cell concentrations after recollection did not differ significantly compared to the original suspension (paired *t*-test, *t* = 0.40, *df* = 14, *p* = 0.70), which confirmed that the dried cells were successfully washed off the surface. After drying and suspension in the respective salt solution, the samples were prepared for analysis by flow cytometry to assess the fraction of live and dead cells as a function of initial salt concentration, RH and droplet size. Droplet drying was also performed on glass slides which confirmed that there was no effect of the material on cell survival (data not shown).

A simplified approximation of the drying rate for sessile droplets with a contact angle of <90°, which is valid for glass and plastic surfaces, was calculated using an equation proposed by Birdi et al. (1989):

(1) $$-\frac{dm}{dt}=4\pi R_{S}D(c_{S}-c_{\infty })$$

where *dm/dt* is the change in mass over time, *R_S_* is the radius of the droplet, *D* is the vapor diffusion coefficient (here for water), *c_S_* is the vapor concentration at the surface of the droplet and *c_∞_* is the vapor concentration in the surrounding air (the RH). The drying time was then plotted for droplets in the size range of 1–80 μL.

### Bacterial Aerosolization From the Bubble Tank

Aerosolization by bubble bursting from aquatic environments was simulated with a custom-made bubble tank. The bubble tank is described in detail in King et al. (2012). Briefly, the tank is a 17 L stainless steel cylinder that was filled with 10 L of MilliQ water (Figure 1B). Bubbles were produced by aeration of the liquid through a stainless-steel diffuser placed at the bottom of the tank. The flowrate of the aeration air, 5 L min^-1^, and the air that passes through the headspace, 10 L min^-1^, were controlled by two mass flow controllers (MFC). The airflow through the headspace of the tank ensured that no ambient air was drawn into the headspace. To prepare the experiment, the tank was filled with MilliQ water (<3 ppb TOC, 18.2 MΩ cm). Thereafter, a suspension of washed bacterial cells was added, the tank was closed and aeration was started. Aeration promoted a rapid and efficient mixing of the bacterial suspension. The bacterial concentration in the tank was ∼5 × 10^7^ cells mL^-1^. Aerosolized bacteria were collected into phosphate-buffered saline (PBS) using a BioSampler (SKC Inc.) with a sampling flow rate of 12.5 L min^-1^. An optical particle size spectrometer (OPS, TSI model 3330, 1 L min^-1^) was used to measure the size distribution of the aerosolized particles in the size range 0.3–10 μm (in 16 size bins). A scanning mobility particle sizer (SMPS, TSI model 3936, sample flow 1 L min^-1^) was used to measure aerosol particle size distributions in the size range 10–512 nm (Supplementary Figure S1). The RH was measured (Rotronic HC2-S) in the headspace in the beginning and in the end of each experiment and varied between 78 and 82%. The bubble tank experiment was performed in six replicates, each 3 h long. A pilot experiment using 3.5 wt% sea salt solution resulted in a high background concentration of aerosolized salt particles at bacterial cell sizes, which made it impossible to distinguish the aerosolized bacteria by the OPS (Supplementary Figure S2). Based on these results we decided to use MilliQ water as the solvent for all bubble tank experiments.

### Bacterial Aerosolization at Varying RH

The effects of drying at different RH on survival of airborne bacteria were assessed using the experimental set up as shown in Figure 1C. Bacterial cells (∼10^9^ cells mL^-1^) were suspended in 0.1 wt% NaCl solution and aerosolized with a sparging liquid aerosol generator (SLAG, CH Technologies) into a humidity conditioned flow tube. In short, a bacterial solution was delivered onto a porous disk by a syringe pump at a rate of 1 mL min^-1^. Simultaneously, an airflow was led through the porous disk, thereby producing bubbles in the bacterial cell suspension that burst and generated droplets containing bacterial cells. From here, the droplets were led into a 1 m long stainless steel flow tube with an inner diameter of 6 cm, at a flow rate of 8 L min^-1^ and mixed with a dilution airflow of 30 L min^-1^. The overall residence time of the cells in the tube was about 4.5 s. The dilution air was adjusted to the desired RH of 10–20, 30–40, 60, and 90%, respectively. The aerosol size distribution in the size range 0.3–10 μm (in 16 size bins) was monitored at the end of the flow tube with an OPS (model 3330, TSI Inc.), and the RH was monitored with a Rotronic probe (model HC2-S). The aerosol particles were collected into 20 mL of 0.1 wt% NaCl solution by a BioSampler (SCK Inc.), which was operated at 12.5 L min^-1^.

The drying time of droplets in air depends on the RH in the surrounding air as well as on droplet size and the solute concentration in the liquid constituting the droplet. The rate of drying for a droplet of pure water can be described by the following equation (Kulkarni et al., 2011):

(2) $$\frac{d(d_{p})}{dt}=\frac{4D_{v}M}{\rho_{p}d_{p}R}\left(\frac{p_{\infty }}{T_{\infty }}-\frac{p_{d}}{T_{d}}\right)\;,\;\;\;for\;d_{p}>\lambda$$

where λ is the mean-free path of the surrounding gas (at standard conditions, λ = 0.066 μm), *D_v_* is the diffusion coefficient of the vapor molecules (here for water), *M* is the molecular weight (for water vapor in air), ρ_p_ is the density of the droplet liquid, *d*_p_ is the initial droplet diameter and *R* is the universal gas constant. Evaporation of water is caused by difference in vapor pressure in the surrounding environment *p_∞_*, at temperature *T_∞_*, and vapor pressure at the droplet surface *p_d_* at temperature *T_d_*. By integration of Equation (2), the time of evaporation of a droplet can be calculated (Equation 3):

(3) $$t=\frac{R\rho_{p}d_{p}^{2}}{8D_{v}M\left(\frac{p_{d}}{T_{d}}-\frac{p_{\infty }}{T_{\infty }}\right)}\mathrm{for}\;\mathrm{initial}\;d_{p}>2\mu m$$

### Cell Survival Assessed by Flow Cytometry

The viability state of the cells was analyzed by flow cytometry using a NovoCyte ACEA (Biosciences Inc., LOD: 0.2–50 μm cell size). A 488 nm laser was used for excitation and 530/30 nm and 615/24 nm bandpass filters for emission measurements of green and red fluorescent light, respectively. Threshold was set on a forward scatter of 500 and the analysis was performed at a flow-rate of 14 μL min^-1^. The *Bac*Light^TM^ kit (Life technologies, Thermo Fisher) was used to determine the proportion of viable cells by assessing membrane integrity of the cells (Stocks, 2004). SYTO9 stains the nucleic acids of all cells and PI can only enter cells with a disrupted cell membrane, due to its larger size and charge. The samples were either stained with concentration ratios of SYTO9 (5 mM) and propidium iodide (PI) (30 mM) of 1:1 (1.5 and 1.5 μL), 1:2 (0.5 and 1.0 μL), or 1:3 (0.5 and 1.5 μL) in a total volume of 1 mL. Stained samples were incubated for 15 min in dark before being analyzed by flow cytometry. In order to correct for the fluorescence spillover between the bandpass filters, a compensation matrix was determined using samples stained with single dyes and applied to the samples stained with both dyes. Unstained samples were used as controls in every experiment. Samples treated with 5 mM EDTA, which permeabilizes the outer membrane (Berney et al., 2007), were used as controls for dead cells (data not shown). We used Flowjo software for data analysis (V10.0.7, Ch 2, Flowjo, LLC 2013-2016).

### Cell Cultivability

Cultivability on agar plates was used as an additional measure of viability in order to compare the outcome of our study with earlier studies. Ten-fold serial dilutions were prepared for each sample and 100 μL of each dilution was plated on R2A nutrient agar plates in triplicates and incubated for 36–48 h at room temperature prior to evaluation. The number of colony-forming units (CFU) on R2A was counted and normalized by the cell concentration determined by flow cytometry.

### Cell Sorting After Aerosolization

We evaluated the cultivability of the different cell populations observed by flow cytometry by employing a combination of fluorescence activated cell sorting and cultivation. Thus, cells collected after aerosolization by SLAG in air with 20% RH were stained with the *Bac*Light^TM^ kit and sorted using FACSAria^TM^ III (BD Biosciences, United States) into 50 μL PBS. The different populations were diluted to 50,000 cell mL^-1^ and their cultivability was assessed. Triplicate experiments were run and evaluated.

### Visualization of Cells by Transmission Electron Microscope

Aerosolized cells collected from the humidity conditioned flow tube and non-aerosolized controls were fixed with 4% paraformaldehyde and stored at 4°C until analyzed. The fixed cell pellets were rinsed in PBS three times for 10 min each before preparation for TEM inspection. Copper grids with a 200 square mesh, coated with a carbon film (Electron Microscopy Sciences, Hatfield, PA, United States) were freshly glow-discharged (PELCO easiGlow^TM^ Glow Discharge Cleaning System, Ted Pella, CA., United States) before use. A 3 μL droplet of the fixed sample was placed on a copper grid and cells were left to settle for 30 s. Thereafter, the liquid was carefully removed using filter paper. To increase the imaging contrast, a 2 μL droplet of 2% uranyl formate was added onto the grids and dried using filter paper. Finally, the samples were washed twice with 2% uranyl formate. After air-drying, TEM images were taken using a FEI Tecnai Spirit electron microscope (FEI Inc., Oreg., United States) with a TWIN lens operating at 120 kV. Images were collected with a Tvips TemCam F416 CMOS camera.

### Statistical Analysis

Aerosolization was performed at six different RHs but to obtain six replicates per group, data from 10 to 20% RH and 30 to 40% RH were pooled into two groups (n_slag_= 24). One sample was excluded due to technical issues. The bubble tank experiment was performed in six replicates (n_bubbletank_= 6, n_aerosol_= 29). The surface drying experiment was performed in triplicates at four RHs (15, 30, 60, 80%, n_surface_= 12) and with five different droplet sizes (1, 5, 10, 25, and 75 μL, n_size_= 15). Surface drying was performed in nine solutions with different salt concentration (MilliQ water, 0.1 wt% NaCl, 0.9 wt% NaCl and 0.5, 1.0, 1.5, 2.0, 2.5, and 3.5% sea salt) with triplicate measurements repeated on two occasions. For four surface drying experiments (MilliQ, 0.5, 1.5, and 2.0% sea salt) only triplicate measurement could be considered (n_salt_= 42). Mean values and standard error of mean was used to describe the obtained results and non-parametric statistical tests were used to evaluate correlations (Spearman’s rho) and differences between groups (Mann–Whitney *U*-test).

## Results and Discussion

In this study, we evaluated the effect of aerosolization and drying on *P. syringae* cells by flow cytometry. Drying of cells in small aerosolized droplets, simulating wet environments, resulted in significantly higher survival than drying of cells in large droplets deposited on a surface, simulating dry environments (*p* < 0.001). Cell survival was also found to increase with decreasing RH (*p* < 0.01), and in the surface drying experiment, with increased salt concentrations (*p* < 0.001).

### Defining Stages of Cell Damage

**FIGURE 2:**
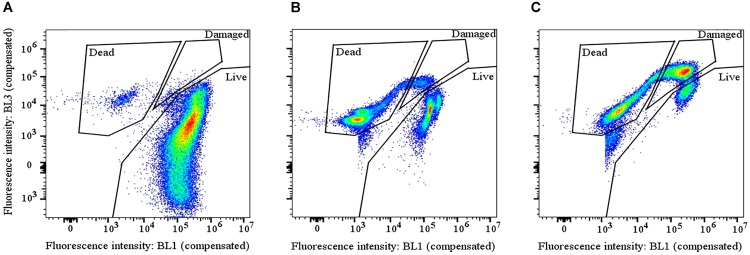
The gating strategy for analysis of flow cytometry results. Dot plots show how live, damaged and dead cell populations were defined based on the fluorescence intensity in the blue laser 1 detector (SYTO9, 530/30 nm) and blue laser 3 detector (PI, 615/24 nm). A representative example was taken from **(A)** cells suspended in 0.1% NaCl prior to aerosolization, **(B)** cells after aerosolization at 40% RH, and **(C)** cells after aerosolization at 60% RH.

Flow cytometry analysis of aerosolized cells revealed the presence of three cell populations based on the fraction of SYTO9 and PI dyes bound to intracellular DNA. We termed the cell populations “live,” “damaged,” and “dead” (Figure 2) in agreement with Lehtinen et al. (2004). The cells constituting the live population did not contain PI, indicating that their cell membranes were intact. Cells belonging to the damaged population contained some PI (fluorescence > 10^4^), indicating a partly damaged cell membrane while cells in the dead population were dominated by PI fluorescence, due to a high degree of cell membrane damage (Stocks, 2004; Supplementary Figures S1–S7).

In order to investigate the cultivability in all three populations, an experiment was performed where live, damaged and dead cells were sorted and each population was cultivated separately. From this experiment only the live population showed cultivability, thus the damaged population was considered non-viable. Only a small fraction (<5%) of the live cells were cultivable, which could either be due to damaging effects of the staining molecules, mechanical damage imposed during cell sorting or the VBNC state of some cells.

From the bubble tank aerosolization experiment, we observed that despite the fact that no live cells were observed, some cells could be cultivated, after a prolonged incubation time (>48 h). This observation indicates that damaged cells were likely not all dead. In addition, damaged cells induced after cell suspension in MilliQ water, could recover and shift back to the live population after being rehydrated in PBS. PI uptake by damaged cells could be explained by peptidoglycan instability in the cell wall, which causes misleading uptake of PI (Shi et al., 2007). Even though the damaged population was not cultivable in the cell sorting experiment, our observations suggested that some damaged cells are VBNC rather than dead. Both live and damaged populations are shown for all experiments in Supplementary Figures S8–S10.

### The Effect of Relative Humidity on Cell Survival

**FIGURE 3:**
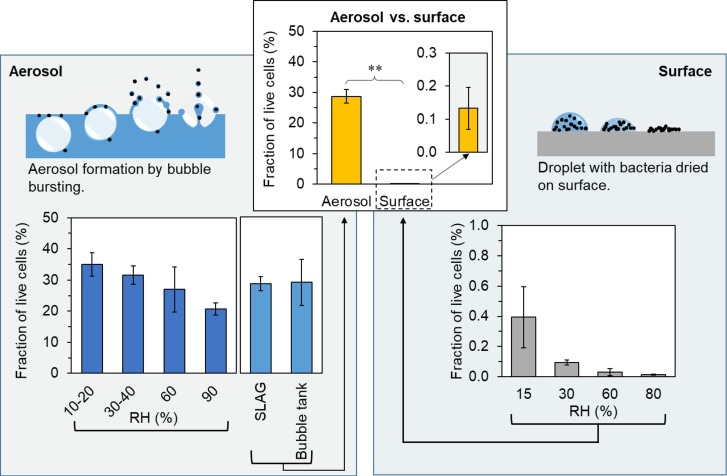
Fraction of live cells after aerosolization and surface drying in air with different RH. Upper central graph: Average fraction of healthy and damaged cells after aerosolization (*n* = 29) and surface drying (*n* = 12). The bar height is the mean of n samples and the error bars represent the standard error of mean (SEM). Left panel: Illustration of bubble bursting together with the flow cytometry results from aerosolization at different RH by SLAG (10–20, 30–40, 60, 90% RH), and a comparison of the SLAG and the bubble tank. Right panel: Illustration of a droplet drying on a surface together with the flow cytometry results from surface drying in air with different RH (15, 30, 60, and 80% RH). ^∗∗^ indicates significance between groups with *p* < 0.001.

**FIGURE 4:**
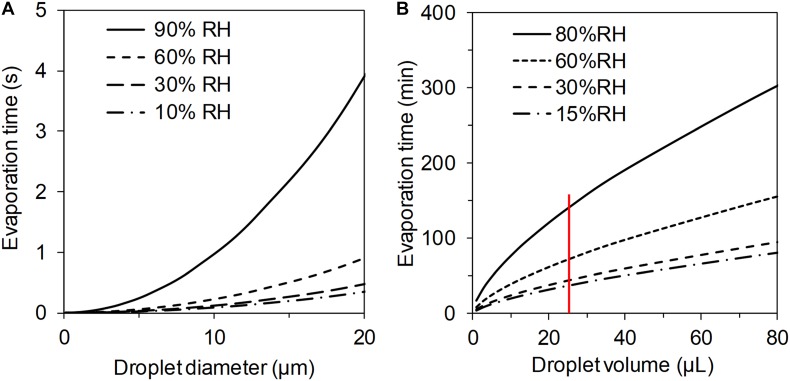
Calculated evaporation times using **(A)** equation 3 for airborne droplets of pure water at different relative humidity (at room temperature). All droplets below 20 μm in diameter, which was all of the aerosolized droplets, would have time to evaporate droplet water around the bacterial cell in the aerosolization setup even at high (90%) RH. **(B)** Calculated drying times for sessile droplets (with a contact angle <90°) on a surface at room temperature using equation 1. The red line indicates the drying times for the surface drying experiment.

The fractions of live cells after both aerosolization with SLAG and surface drying experiments showed significant inverse correlation with RH (Spearman’s rho = -0.56, *p* < 0.01 for aerosolization and rho = -0.73, *p* < 0.01 for surface drying Figure 3). The different levels of RH resulted in different drying times for both aerosolized and surface droplets. In both experiments, the main stress for the cells was drying, although aerosolized cells were also exposed to mechanical forces during emission. The drying times for aerosol (pL) droplets and surface dried (μL) droplets were on the scale of seconds and hours, respectively. Approximate drying times for pure water droplets in aerosol and on surfaces, respectively, were calculated and plotted in Figure 4A,B. The low salt concentration (0.1 wt% NaCl) that was used in these experiments was considered to have a negligible effect on the drying time, but it may have affected the dryness of the particle due to the physiochemical properties of NaCl. Drying of a NaCl particle follows an efflorescence curve, which describes gradual dehydration of the particle with decreasing RH to a certain efflorescence relative humidity (ERH) where all water is evaporated and the salt forms crystals. For NaCl particles, the ERH is at 45% RH (Biskos et al., 2006). This means that NaCl that is left on the cell surface after drying either retains some liquid water (above the ERH) or is completely dry and hence in crystal form (below the ERH). A possible explanation for the higher survival at lower RH could be (i) the shorter drying times and (ii) completely dry salt particles, which result in a lower osmotic stress for the cell.

**FIGURE 5:**
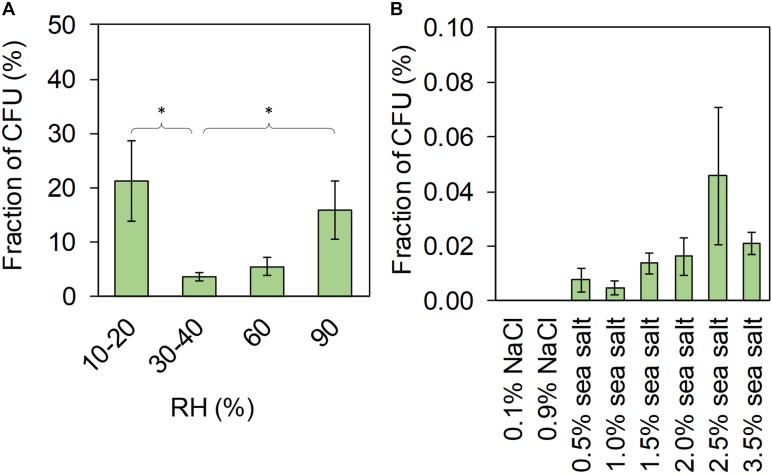
Results from cultivation showing the fraction of CFU per total cells (total cell concentration from flow cytometry) for **(A)** aerosolized cells, **(B)** and surface dried cells. The bar height represents the mean of three replicates and the error bar, the standard error of the mean. ^∗^ indicates significance between groups, *p* < 0.05, using Mann–Whitney *U*-test.

Cultivation of aerosolized cells showed another pattern of survival in relation to RH than the flow cytometry (Figure 5A) and no correlation was found between the CFU and the live cells from flow cytometry. Cultivation results were similar to those found in an earlier study with lower cultivability at intermediate RH and high cultivability at low and high RH (Cox, 1971). We found significant differences between the groups 10–20% and 30–40% RH (Mann–Whitney *U*-test *Z* = –2.4, *p* = 0.015) and between the groups 30–40% and 90% RH (*Z* = –2.4, *p* = 0.017). The survival of aerosolized bacteria at different temperatures and RH has been addressed in a few studies, both from the atmospheric and the indoor air perspective (Dunklin and Puck, 1948; Webb, 1959; Cox, 1966, 1971; Won and Ross, 1966; Marthi et al., 1990; Walter et al., 1990; Lighthart and Shaffer, 1997; Ko et al., 2000; Tang, 2009). Most of these studies report a change in survival at different RH, but the conclusions are inconsistent and vary almost as much as the methodologies that have been applied. A higher fraction of live cells after surface drying at lower RH agrees with the findings by Bauermeister et al. (2011), where a higher RH (∼33%) also was found to be more damaging to cells of non-spore-forming bacteria than lower RH (∼4%). Marthi et al. (1990) concluded that lower air temperature (and higher RH, due to unchanged water vapor content) increased the survival of aerosolized *P. syringae*, possibly due to a decrease in metabolic activity. However, considering the large droplets they used (130 and 450 μm in diameter), it is likely that they did not dry out at the high RH (77%), thus not exposing cells to drying stress. All these studies have based their results on cultivability, where VBNC cells are not considered. Hence, our study advances the understanding of how drying effects cell survival by using the cultivation independent approach based on live/dead staining and flow cytometry. Using the cultivation independent approach, we found that faster drying rates at low RH and complete removal of liquid water promote survival of bacterial cells.

### Comparison of Cell Survival in Aerosolization and Surface Drying

The average fraction of live cells after aerosolization (n_aerosol_ = 29) was significantly higher than the average fraction of live cells after drying on a surface (n_surface_ = 12) (Mann–Whitney *U*-test, *Z* = -4.8, *p* < 0.001) as seen in Figure 3, upper central graph. After aerosolization, the average fraction of live cells assessed by flow cytometry was 29% (range 10–47%), while the fraction of live cells after droplet drying on a surface in air with different RH was 0.1% (range 0–0.8%). There are differences between these two types of experiments that should be considered. Experiments, in which we used the bubble bursting process to simulate aerosolization from wet environments, impose two types of stress on the cells: (i) mechanical stress during the process of bubble bursting and (ii) drying stress. Experiments, in which cells were dried on surfaces to simulate drying in terrestrial environments, only impose the drying stress, while the mechanical stress is absent. Thus, assuming that these two drying methods affect cells in a similar manner, we initially expected that surface drying would have a less detrimental effect on bacterial survival, which was contradicted by our findings. Also, as the surface-dried bacteria were not aerosolized after drying, we can only report the upper limits for the survival after aerosolization from dry environments, as subsequent aerosolization would add additional stress on bacterial cells further affecting their viability. Amato et al. (2007) and Hu et al. (2018), who investigated *in situ* viability of atmospheric airborne cells, found that bacteria in cloud droplets originating from seawater had an increased fraction of cultivable cells compared to cells sampled in clouds that formed over land. Thus, aerosolization from dry terrestrial environments may be a quantitatively important contributor of bioaerosol to the atmosphere but its importance in terms of spreading microbes to new environments may be less important as many cells would have died before reaching a new habitat. In conclusion, our results suggest that aerosolization from liquid and wet environments promotes the survival of bacterial cell compared to the aerosolization from dry environments.

Differences between drying in aerosol and on surface also include potential surface effects and the large differences in droplet size (pL in aerosol compared to μL on surface), which resulted in a large span of drying times. The effect of surface properties (glass and plastic) was evaluated and no difference in cell survival was observed. In addition, an experiment was performed to evaluate the effect of droplet sizes (1, 5, 10, 25, and 75 μL), and thus the drying time, on cell survival. The fraction of live cells was significantly higher in 1 μL droplets compared to all other droplet sizes (Mann–Whitney *U*-test, *Z* = –2.5, *p* = 0.004, Supplementary Figure S11). However, no difference in cell survival was observed for the larger droplet sizes. All in all, our results indicate that smaller droplet sizes, promoting shorter drying times, improve cell survival.

### Effect of Ionic Strength on Cell Viability

**FIGURE 6:**
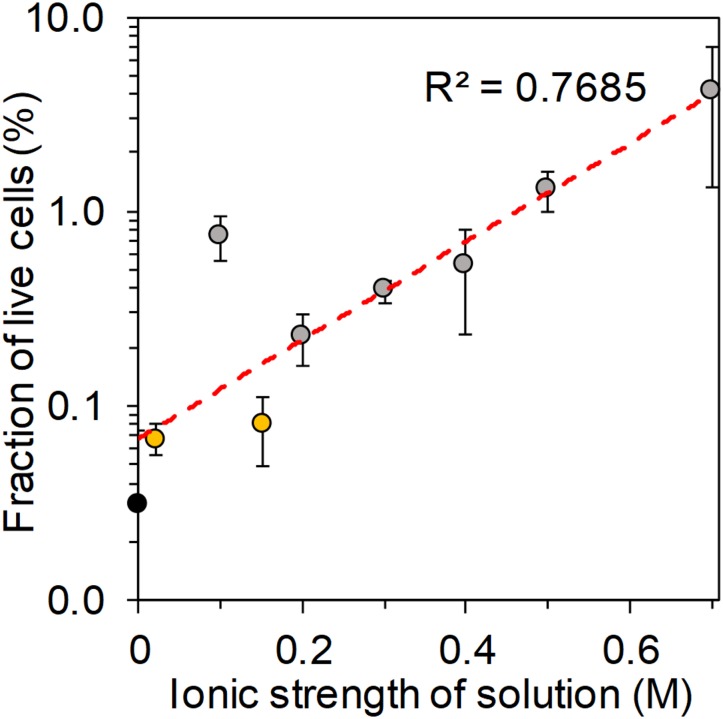
Average fraction of live cells obtained by flow cytometry analysis of the cells suspended in different salt solutions and dried on a surface. Error bars indicate standard error of mean. The colors mark different salt types: black for MilliQ water, yellow for NaCl solutions and gray for sea salt solutions. The red dotted line is an exponential fit to the data points. Note that the y-axis has a logarithmic scale.

The fraction of live cells after surface drying in different salt solutions showed a significant correlation with increasing ionic strength of the tested salt solutions (*p* < 0.001, Spearman’s rho = 0.87, Figure 6). Fractions of cultivable surface-dried cells, shown in Figure 5B, correlated significantly with the fractions of live surface-dried cells (Spearman’s rho = 0.87, *p* < 0.001). We observed a significant correlation between the cultivable cell fraction and an increasing sea salt concentration (Spearman’s rho = 0.66, *p* = 0.005, NaCl excluded). During drying, cells experience osmotic stress as the concentration of extracellular salt solutes progressively increases when water evaporates. In the surface drying experiments, the drying process was on the time scale of minutes to hours, thus the cells had time to respond to the stress by producing protective compounds. Cells can for example regulate the concentration of internal solutes, so called osmoprotectants, in order to prevent changes in cytoplasmic volume (Csonka, 1989). Cell survival was promoted for cells dried in sea salt compared to cells dried in NaCl solutions, indicating that the sea salt mixture contains ions that improve osmoregulation. We suggest that potassium ions, which are often used as primary bacterial response to osmotic stress (Csonka, 1989), may have been involved as osmoprotectants. In the same way, osmoprotectants may be relevant for airborne bacteria, which encounter great variation in osmolality due to repeated condensation-evaporation cycles (Pruppacher and Jaenicke, 1995). In conclusion, we found that cell survival improved (i) at increasing salt concentration and (ii) in presence of sea salt, which contained protective ions.

### Airborne Bacteria Desiccated as Single Cells

**FIGURE 7:**
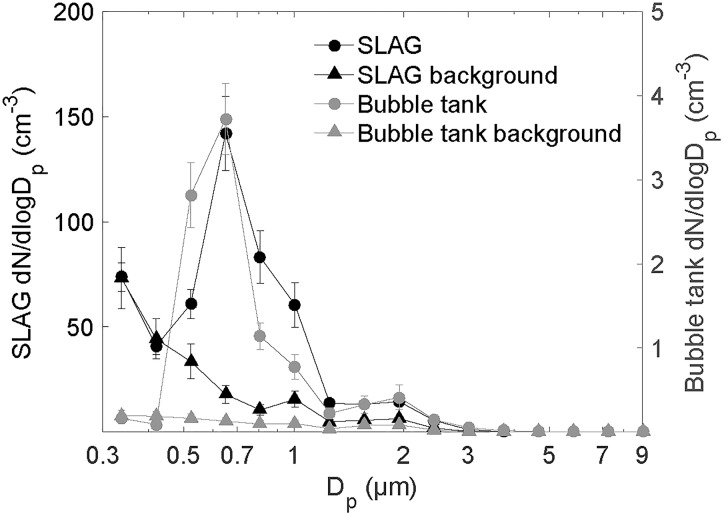
Number size distribution of the aerosol particles generated by SLAG and dried at 10% RH (left y-axis), and by the bubble tank (right y-axis), measured by an OPS. For SLAG, the concentration is a mean value based on 3 replicates (30 min sampling each), and for the bubble tank, the mean is based on one replicate (3 h sampling). The number size distribution of the background NaCl solution for SLAG and MilliQ water for the bubble tank is included (triangular markers).

Aerosolization by both SLAG and the bubble tank generated airborne particles of single bacterial cells. This was concluded based on the aerosol particle size distribution (Figure 7) showing a main peak at 0.7 μm, which is in agreement with the aerodynamic diameter of airborne *Pseudomonas* cells. The second peak at around 2 μm results from the presence of airborne particles containing ≥2 cells. We conclude this based on the observation that the height of this peak decreased when diluting the bacterial suspension (data not shown). Based on the original cell concentration and assumed initial droplet sizes of 1–20 μm, it is likely that each cell containing droplet produced by SLAG has between 1 and 4 cells. The particle size distributions of both SLAG and bubble tank aerosolization are very similar, which was expected as both rely on bubble bursting from bacterial suspensions in low-solute water. No difference in cell survival was observed between aerosolization by the SLAG and the bubble tank. The number concentration of aerosol particles was significantly lower in the headspace of the bubble tank (right y-axis) than in the flow tube connected to SLAG (left y-axis). Figure 7 shows that the number of particles <0.5 μm is higher in the flow tube experiments than in the bubble tank experiments. These are ascribed to salt particles that were aerosolized from the 0.1 wt% NaCl solution. The presence of NaCl in the flow tube experiments could have contributed to increased sizes of particles <0.5 μm at high RH due to the hygroscopicity of NaCl in the particles.

### Transmission Electron Microscopy of Cells Damaged by SLAG Aerosolization

**FIGURE 8:**
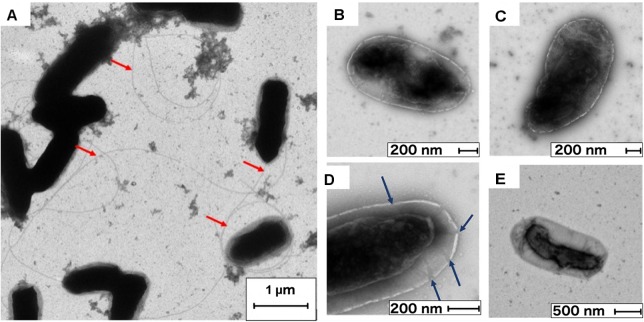
Transmission electron microscopy images of **(A)** healthy cells with intact flagella (flagella marked by red arrows) that had not been aerosolized, and of **(B–E)** cells after aerosolization by SLAG. **(B,C)** Wrinkled cells surfaces, **(D)** porous cell wall (pores marked by blue arrows) and shrunk cytoplasm, and **(E)** detached cytoplasmic membrane and lost flagella.

TEM analysis was used to qualitatively study the membrane integrity and intracellular alteration of *P. syringae* cells before and after aerosolization by SLAG. The images show that aerosolization by SLAG led to cell damages of bacteria in terms of shrunken cytoplasm, porous cell walls and loss of flagella (Figures 8B–E). Before aerosolization, the rod-shaped cells had intact cell walls and cytoplasmic membranes (Figure 8A). After aerosolization by SLAG, a large proportion of the cells had their cytoplasmic membrane detached from the cell wall, which was observed as a shrunken dark-stained cytoplasm (Figures 8B,D). The effects of different drying processes on cells that are described in Potts (1994) show that rapid drying can cause cytoplasm shrinkage, which agrees with our TEM photographs of aerosolized cells. Intact flagella were also less frequent after aerosolization than before, which was interpreted as aerosolization induced cell damage. Visible pores in the cell wall indicate loss of membrane integrity. Mainelis et al. (2002) demonstrated that aerosolization can cause changes in the electrical potential of the cell membrane, which can affect the ion channels and ultimately the membrane integrity. All in all, both TEM and flow cytometry analysis indicated that aerosolization imposes cell wall damage on a large fraction (∼30%) of airborne cells.

## Conclusion

Based on our results, we suggest that:

• Cells that dry on a surface, and that subsequently get aerosolized by wind, have a significantly lower survival rate than cells that get aerosolized from a liquid through the formation of small, fast drying droplets.
• Two factors increase the survival probability of bacterial cells: high concentration of salt in the solution and low RH. Both factors facilitate rapid drying, which we suggest being key to successful airborne dispersal.
• The mechanisms underlying our observation were not identified and need to be elucidated in future studies.
• And finally, the context in which bacterial cells get aerosolized is essential for their further life history. As living cells, they can impact the atmosphere through active participation in carbon, nitrogen and water cycles as well as spread into new environments and to new hosts.

## Author Contributions

The study concept and design was developed by SH, MA, JL, and TŠ-T, and the experiments were executed by SH, MA, SC, MS, BR, ML, and TŠ-T. Data analysis was performed by SH, MA, SC, MS, BR, ML, TB, and TŠ-T. All authors were involved in several rounds of critically reviewing the manuscript.

## Conflict of Interest Statement

The authors declare that the research was conducted in the absence of any commercial or financial relationships that could be construed as a potential conflict of interest.
