# Corrigendum: Effect of Aerosolization and Drying on the Viability of *Pseudomonas syringae* Cells

**DOI:** 10.3389/fmicb.2019.02378

**Published:** 2019-10-15

**Authors:** Malin Alsved, Stine Holm, Sigurd Christiansen, Mads Smidt, Bernadette Rosati, Meilee Ling, Thomas Boesen, Kai Finster, Merete Bilde, Jakob Löndahl, Tina Šantl-Temkiv

**Affiliations:** ^1^Ergonomics and Aerosol Technology, Department of Design Sciences, Lund University, Lund, Sweden; ^2^NanoLund, Lund University, Lund, Sweden; ^3^Department of Physics and Astronomy, Stellar Astrophysics Centre, Aarhus University, Aarhus, Denmark; ^4^Microbiology Section, Department of Bioscience, Aarhus University, Aarhus, Denmark; ^5^Atmospheric Physical Chemistry, Department of Chemistry, Aarhus University, Aarhus, Denmark; ^6^Department of Molecular Biology and Genetics, Aarhus University, Aarhus, Denmark

**Keywords:** bioaerosols, aerosolization, *Pseudomonas syringae*, drying, bubble bursting, ice nucleation activity

Bernadette Rosati was not included as an author in the published article. The corrected Author Contributions Statement appears below:

“The study concept and design was developed by SH, MA, JL, and TŠ-T, and the experiments were executed by SH, MA, SC, MS, BR, ML, and TŠ-T. Data analysis was performed by SH, MA, SC, MS, BR, ML, TB, and TŠ-T. All authors were involved in several rounds of critically reviewing the manuscript.”

The authors apologize for this error and state that this does not change the scientific conclusions of the article in any way. The original article has been updated.

