# Supplementary material for: Effect of Aerosolization and Drying on the Viability of Pseudomonas syringae Cells
Source: Front Microbiol. 2018 Dec 18;9:3086. doi: 10.3389/fmicb.2018.03086 (PMC6305290; doi:10.3389/fmicb.2018.03086)
Supplement: Supplementary file 1 [file Data_Sheet_1.PDF]

Supplementary material to the article “Effect of aerosolization and drying on *Pseudomonas syringae* cells”

## 1. Airborne particle number concentration in the head space of the bubble tank

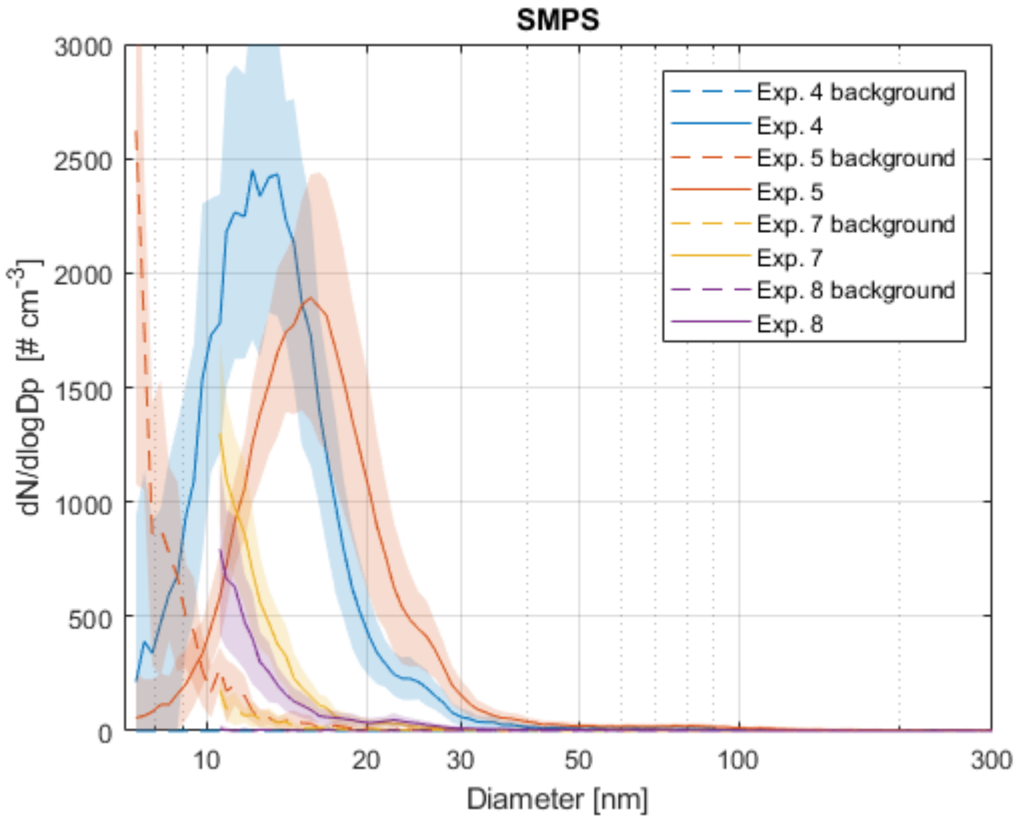


Figure S1. Particle number concentration measured by an SMPS system during four of the bubble tank experiments. Dotted lines are the particle number concentrations from the bubble tank when it was filled with only MilliQ water, and the solid lines are the concentrations when the bacteria had been added.

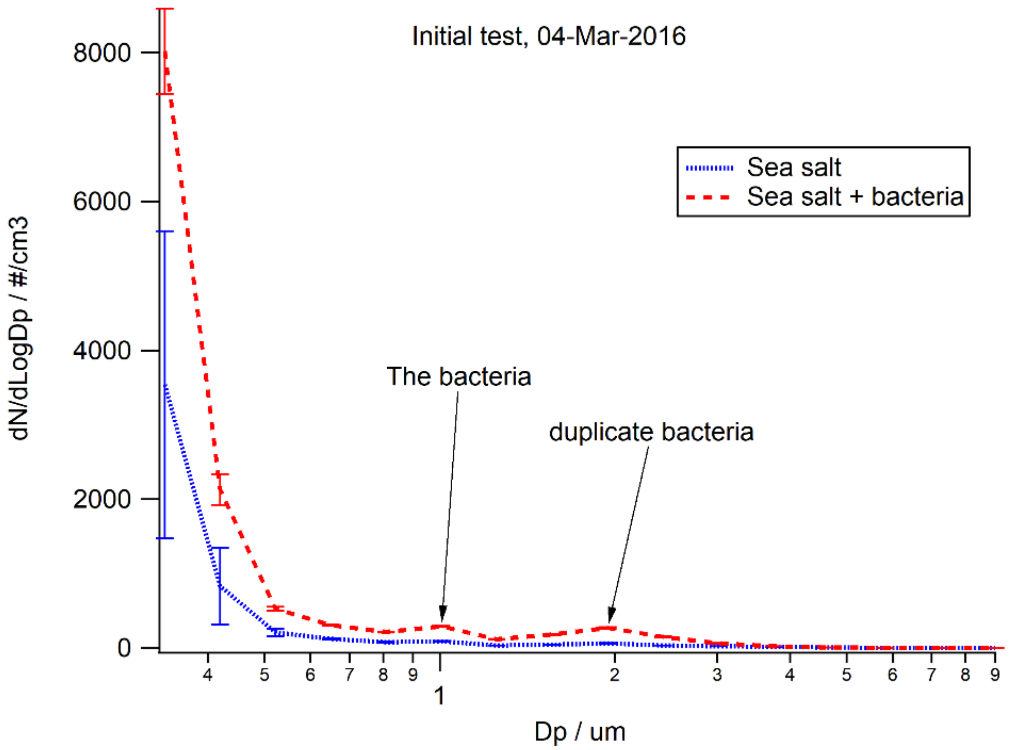


Figure S2. Particle number concentration measured by an OPS in the head space of the bubble tank when using 3.5% sea salt concentration as the bulk suspension liquid. The high number of salt particles made it difficult to distinguish the number of bacteria from the background.

## 2. Gating strategy in the flow cytometry analysis

In figure S1, a general gating example from the aerosolization experiment is shown.

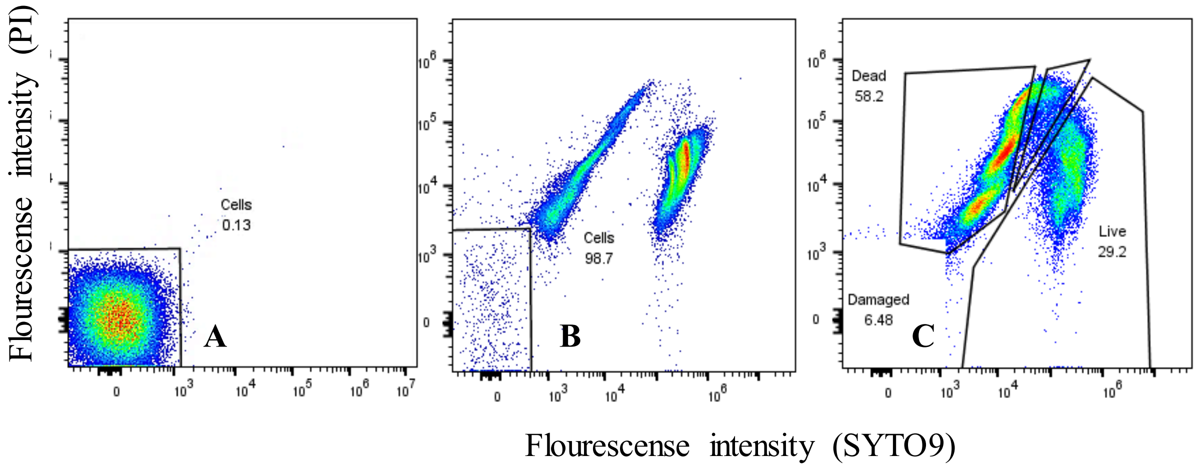

Figure S3. A) A dot plot of an unstained sample showing how gating was applied to define cells (%) and how the background was removed. B) A non-treated double stained sample, resuspended in 0.1% NaCl and the cell proportion is defined (%). C) Within the cell gate, the three cell populations live (%), damaged (%) and dead (%) are defined. The three cell populations in an aerosolized sample.

For each treatment the same strategy was applied based on how the three cell populations dead, damages and healthy were shaped. Below we show a gating example for each experiment, after compensation (see methods) was applied. The x-axis defines the intensity of the live stain (SYTO9, 530/30 nm) the y-axis defines the dead stain, blue laser 3 detector (PI, 615/24 nm). For each experiment an example of a; double stained non-treated sample defining the cell proportion (A), a double stained sample of a non-treated sample (B). A double stained sample of the treated sample (C), is shown.

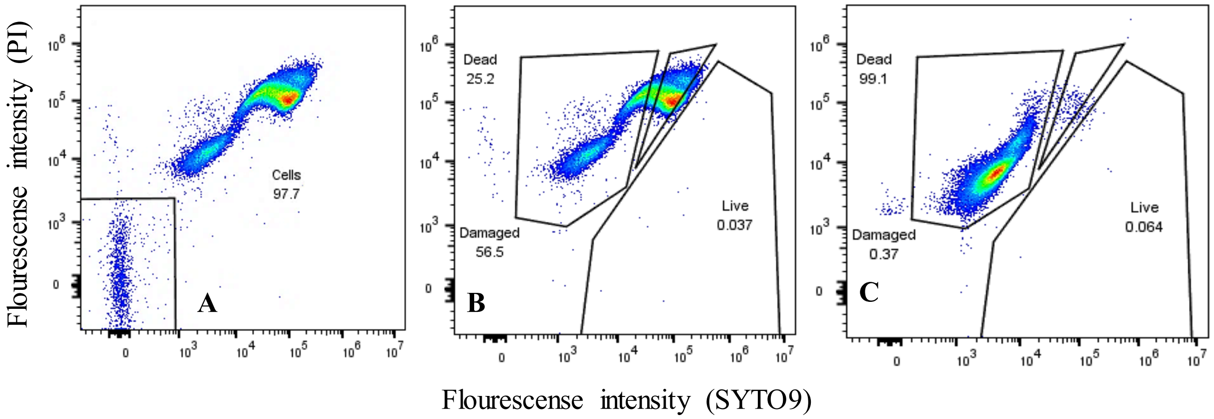


Figure S4. Example of the gates used in the surface drying experiment with different salt concentrations 0-3.5%. A representative sample of cells dried in MilliQ is shown. A) A double stained non-treated sample defining the cell population. B) A double stained non-treated sample defining the three populations. C) A double stained sample of the treated sample.

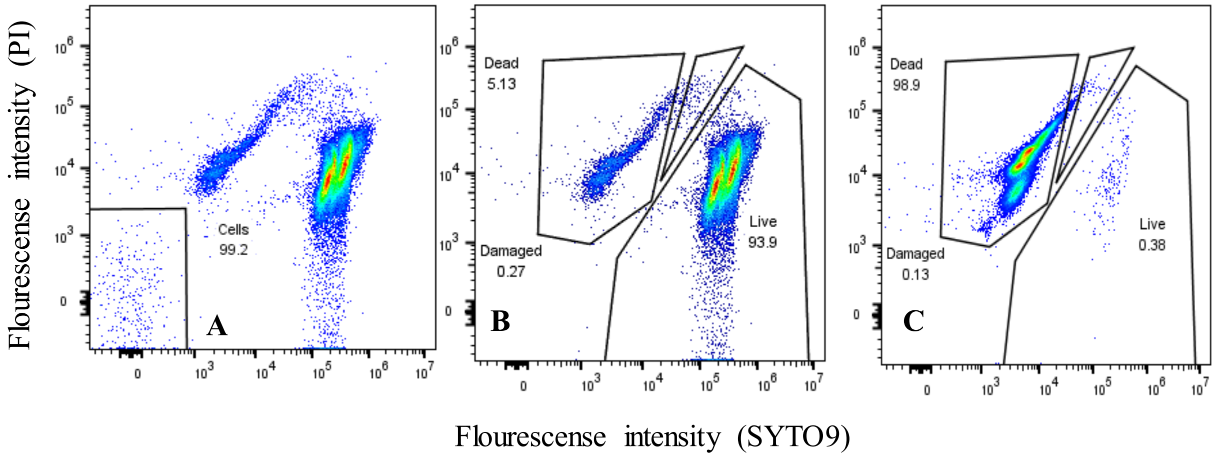


Figure S5. Example of the gates used in the surface drying experiment at different relative humidity (15, 30, 60 and 80% RH). A representative sample dried at 15% RH is shown. A) A double stained non-treated sample defining the cell population. B) A double stained non-treated sample defining the three populations. C) A double stained sample of the treated sample.

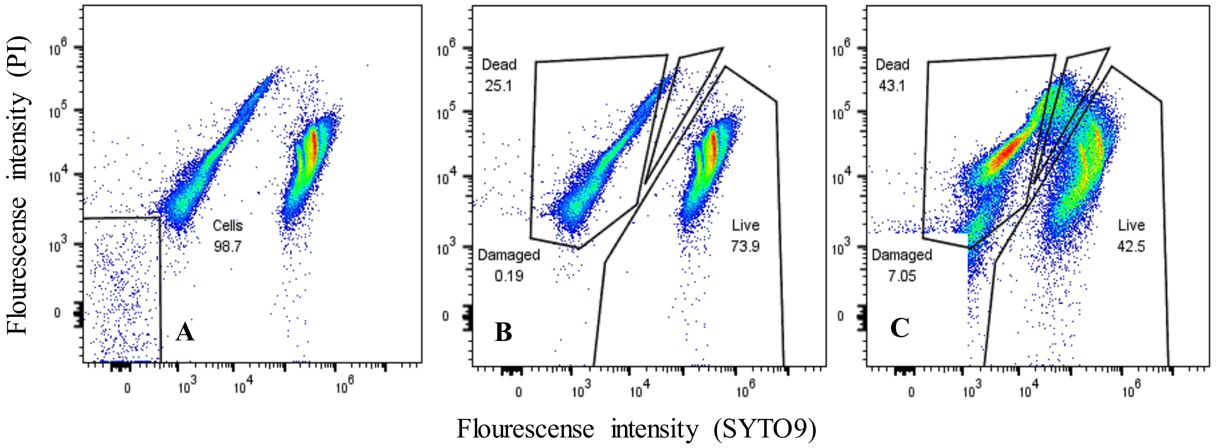


Figure S6. Example of the gates used in the aerosolization experiment at different relative humidity (10, 30, 60 and 90% RH). A representative sample aerosolized in 60% RH is shown. A) A double stained non-treated sample defining the cell population. B) A double stained non-treated sample defining the three populations. C) A double stained sample of the treated sample.

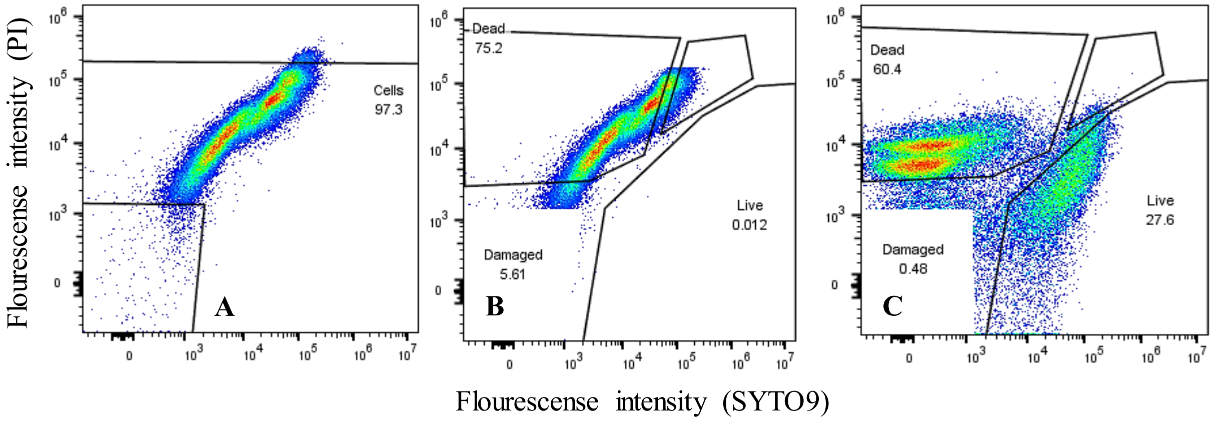


Figure S7. Example of the gates used in the aerosolization experiment with the bubble tank. A) A double stained non-treated sample defining the cell population. B) A double stained non-treated sample defining the three populations. C) A double stained sample of the treated sample.

## 3. Fractions of live and damaged cells

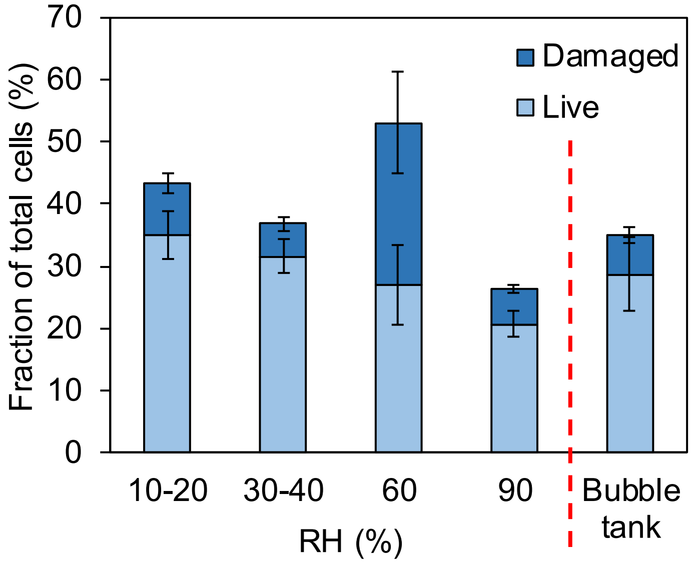


Figure S8. Average fraction of live and damaged cells after aerosolization by the SLAG in different RH and the bubble tank. The error bars represent the standard error of the mean.

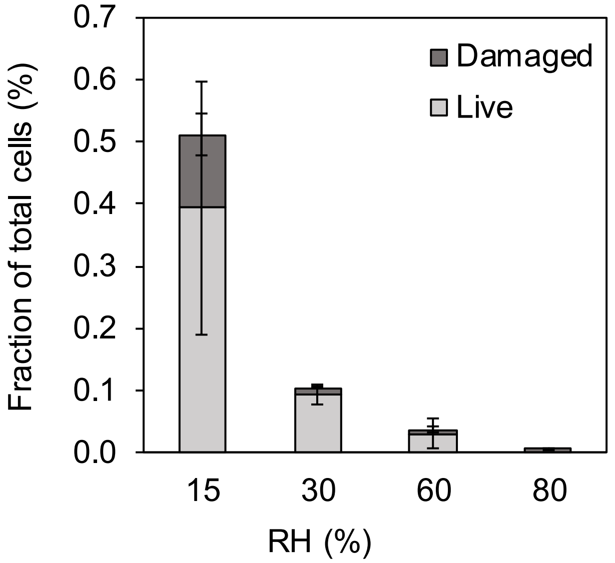


Figure S9. Average fraction of live and damaged cells after surface drying in air with different relative humidity. The error bars represent the standard error of the mean.

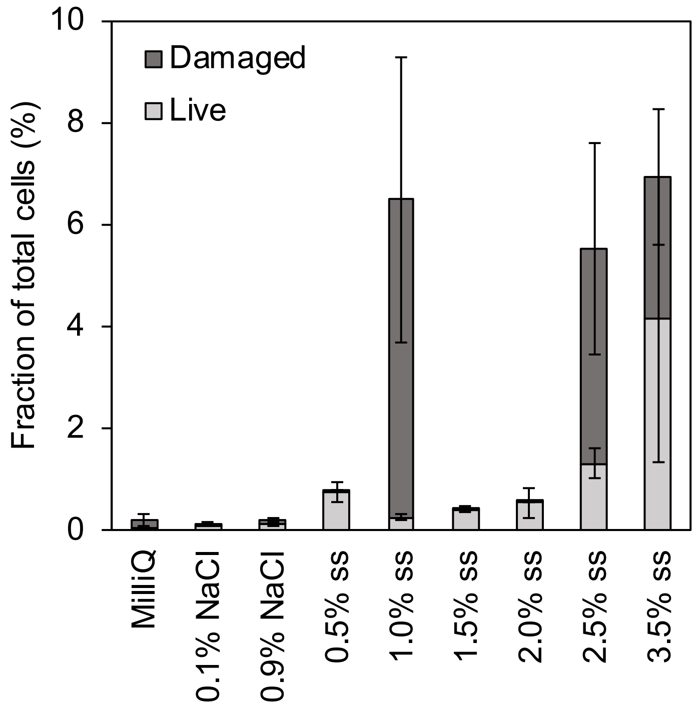


Figure S10. Average fraction of live and damaged cells after surface drying in different salt solutions (ss = sea salt). The error bars represent the standard error of the mean.

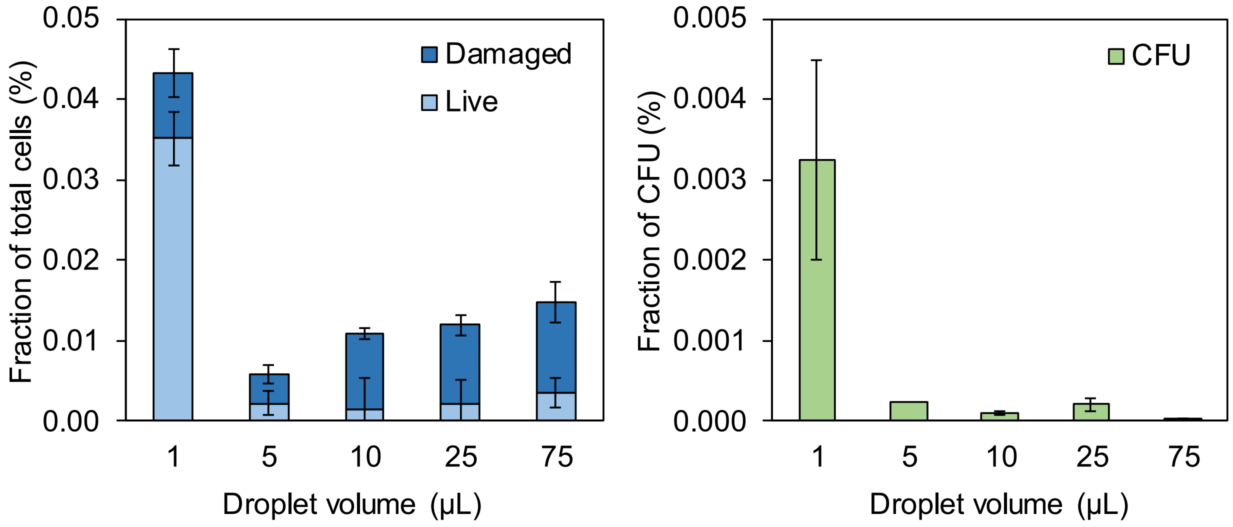


Figure S11. Left, average fraction of live and damaged cells after surface drying of droplets of different volume size measured by flow cytometry, and right, the fraction of CFUs from the same experiment. The error bars represent the standard error of the mean.
